# Supplementary material for: The African Swine Fever Virus with MGF360 and MGF505 Deleted Reduces the Apoptosis of Porcine Alveolar Macrophages by Inhibiting the NF-κB Signaling Pathway and Interleukin-1β
Source: Vaccines (Basel). 2021 Nov 22;9(11):1371. doi: 10.3390/vaccines9111371 (PMC8622997; doi:10.3390/vaccines9111371)
Supplement: Supplementary file 1 [file vaccines-09-01371-s001.zip › vaccines-1374545-supplementary.pdf]

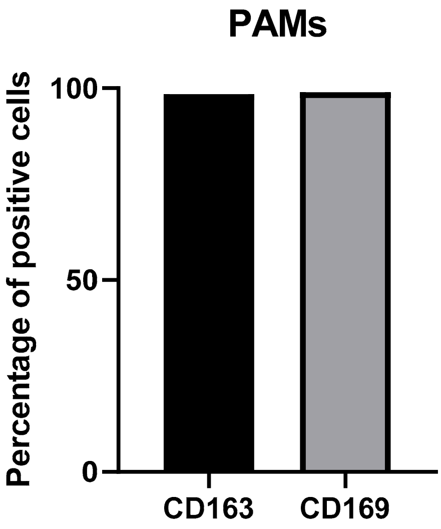

Figure S1. Detection of PAMs cell purity. Expression profiles of these receptors were also determined by flow cytometry showing intense fluorescence staining for all markers as expected for primary tissue macrophages. The positive rate of CD163 on the cell surface was 98.48%, and the positive rate of CD169 was 98.92%.

A

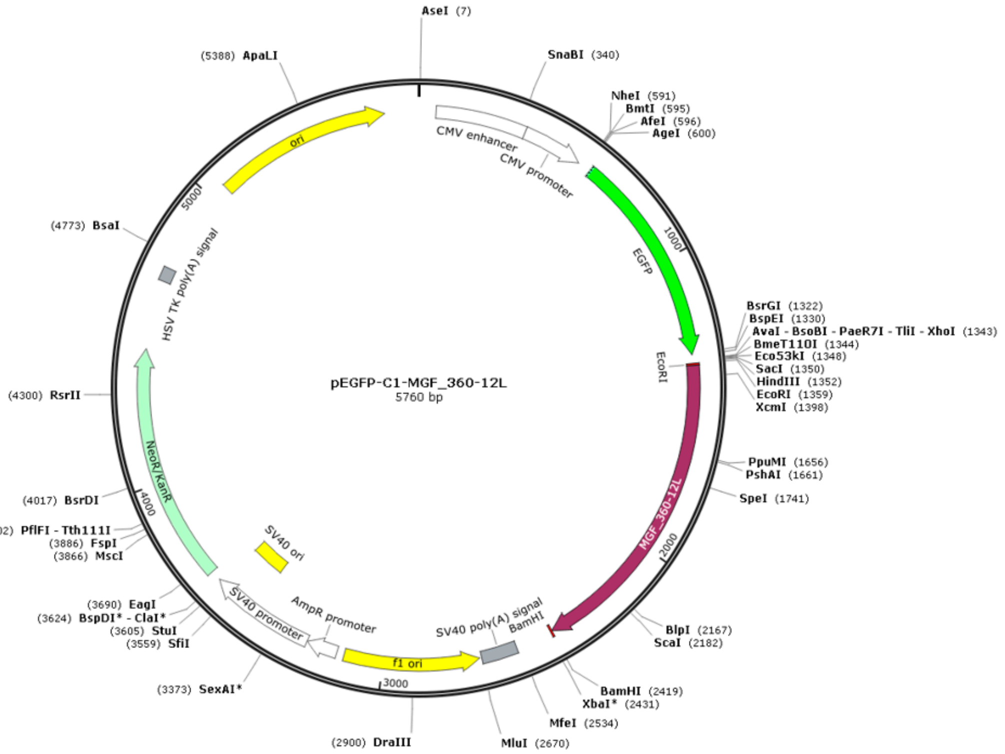

B

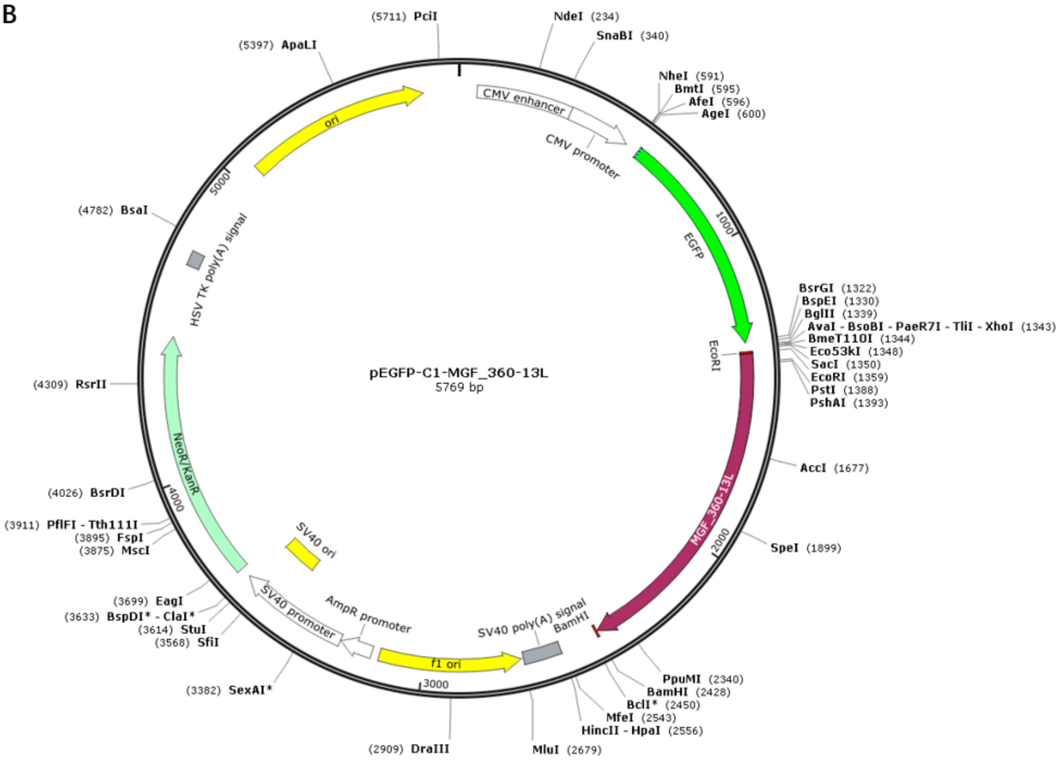
pEGFP-C1-MGF_360-13L
5769 bp
(5711) PciI
NdeI (234)
SnaBI (340)
NheI (591)
BmtI (595)
AfeI (596)
AgeI (600)
BsrGI (1322)
BspEI (1330)
BglII (1339)
AvaI - BsoBI - PaeR7I - TliI - XhoI (1343)
BmeT110I (1344)
Eco53kI (1348)
SacI (1350)
EcoRI (1359)
PstI (1388)
PshAI (1393)
AccI (1677)
SpeI (1899)
PpuMI (2340)
BamHI (2428)
BclI* (2450)
MfeI (2543)
HincII - HpaI (2556)
MluI (2679)
(2909) DraIII
(3382) SexAI*
(3568) SfiI
(3614) StuI
(3633) BspDI* - ClaI*
(3699) EagI
(3875) MscI
(3895) FspI
(3911) PflFI - Tth111I
(4026) BsrDI
(4309) RsrII
(4782) BsaI
(5397) ApaLI
CMV enhancer
CMV promoter
EGFP
EcoRI
MGF_360-13L
BamHI
SV40 poly(A) signal
f1 ori
AmpR promoter
SV40 promoter
SV40 ori
NeoR/KanR
HSV TK poly(A) signal
ori
1000
2000
3000
4000
5000

C

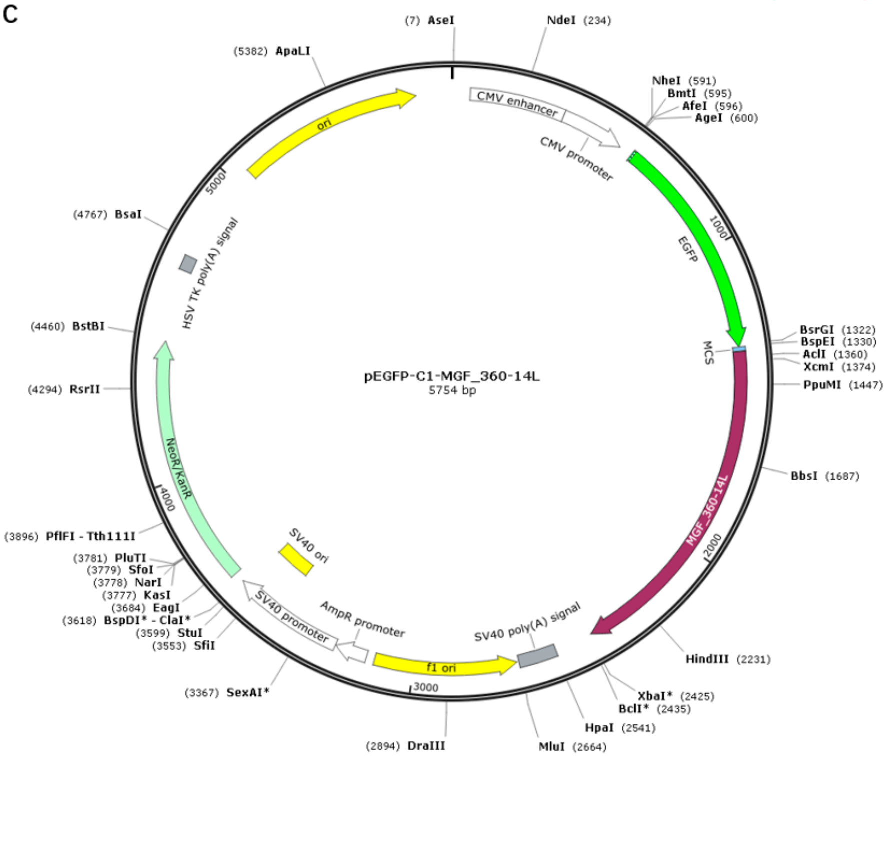
pEGFP-C1-MGF_360-14L
5754 bp
(7) AseI
NdeI (234)
NheI (591)
BmtI (595)
AfeI (596)
AgeI (600)
BsrGI (1322)
BspEI (1330)
AclI (1360)
XcmI (1374)
PpuMI (1447)
BbsI (1687)
HindIII (2231)
XbaI* (2425)
BclI* (2435)
HpaI (2541)
MluI (2664)
(2894) DraIII
(3367) SexAI*
(3553) SfiI
(3599) StuI
(3618) BspDI* - ClaI*
(3684) EagI
(3777) KasI
(3778) NarI
(3779) SfoI
(3781) PluTI
(3896) PflFI - Tth111I
(4294) RsrII
(4460) BstBI
(4767) BsaI
(5382) ApaLI
CMV enhancer
CMV promoter
EGFP
MCS
MGF_360-14L
SV40 poly(A) signal
f1 ori
AmpR promoter
SV40 promoter
SV40 ori
NeoR/KanR
HSV TK poly(A) signal
ori
1000
2000
3000
4000
5000

D

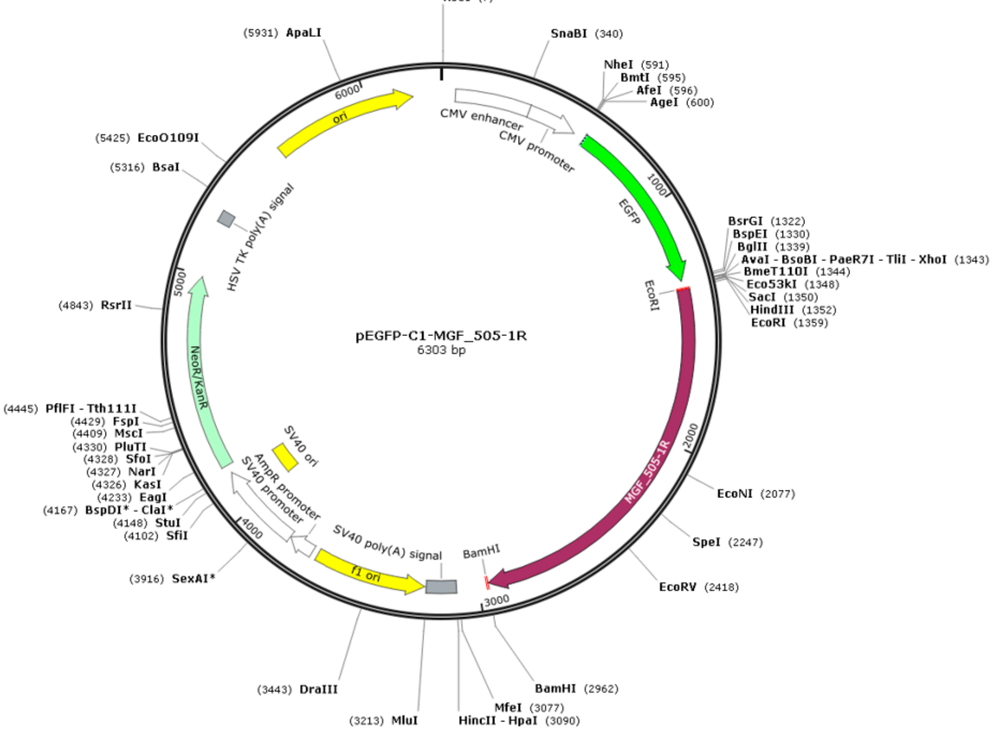
pEGFP-C1-MGF_505-1R
6303 bp
AseI (7)
SnaBI (340)
NheI (591)
BmtI (595)
AfeI (596)
AgeI (600)
BsrGI (1322)
BspEI (1330)
BglII (1339)
AvaI - BsoBI - PaeR7I - TliI - XhoI (1343)
BmeT110I (1344)
Eco53kI (1348)
SacI (1350)
HindIII (1352)
EcoRI (1359)
EcoNI (2077)
SpeI (2247)
EcoRV (2418)
BamHI (2962)
MfeI (3077)
HincII - HpaI (3090)
(3213) MluI
(3443) DraIII
(3916) SexAI*
(4102) SfiI
(4148) StuI
(4167) BspDI* - ClaI*
(4233) EagI
(4326) KasI
(4327) NarI
(4328) SfoI
(4330) PluTI
(4409) MscI
(4429) FspI
(4445) PflFI - Tth111I
(4843) RsrII
(5316) BsaI
(5425) EcoO109I
(5931) ApaLI
CMV enhancer
CMV promoter
EGFP
EcoRI
MGF_505-1R
BamHI
SV40 poly(A) signal
f1 ori
AmpR promoter
SV40 promoter
SV40 ori
NeoR/KanR
HSV TK poly(A) signal
ori
1000
2000
3000
4000
5000
6000

E

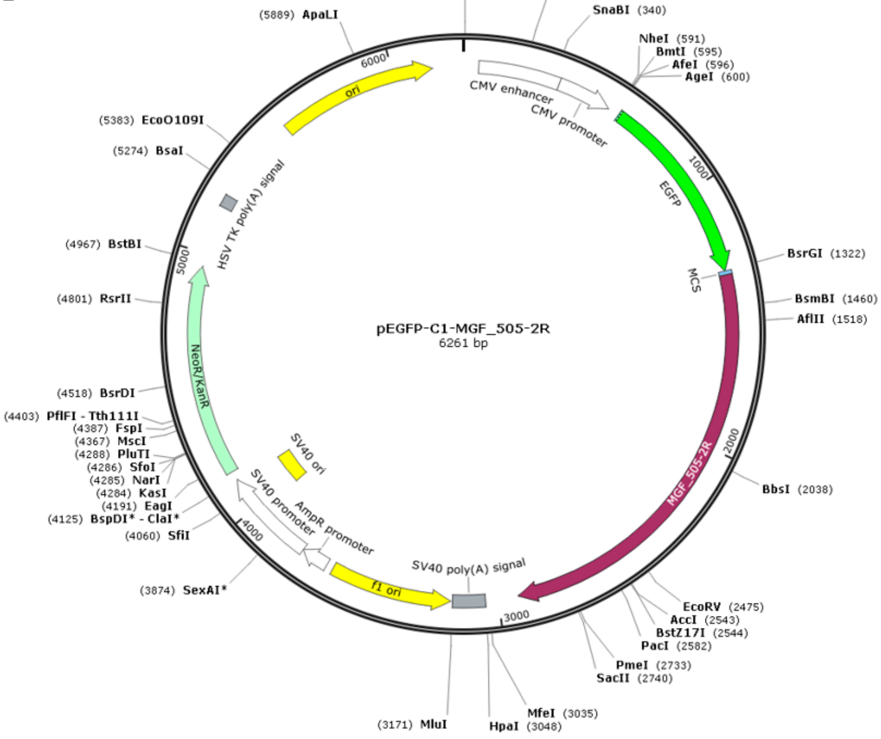
pEGFP-C1-MGF_505-2R
6261 bp
(7) AseI
NdeI (234)
SnaBI (340)
NheI (591)
BmtI (595)
AfeI (596)
AgeI (600)
BsrGI (1322)
BsmBI (1460)
AflII (1518)
BbsI (2038)
EcoRV (2475)
AccI (2543)
BstZ17I (2544)
PacI (2582)
PmeI (2733)
SacII (2740)
MfeI (3035)
HpaI (3048)
(3171) MluI
(3874) SexAI*
(4060) SfiI
(4125) BspDI* - ClaI*
(4191) EagI
(4284) KasI
(4285) NarI
(4286) SfoI
(4288) PluTI
(4367) MscI
(4387) FspI
(4403) PflFI - Tth111I
(4518) BsrDI
(4801) RsrII
(4967) BstBI
(5274) BsaI
(5383) EcoO109I
(5889) ApaLI
CMV enhancer
CMV promoter
EGFP
MCS
MGF_505-2R
SV40 poly(A) signal
f1 ori
AmpR promoter
SV40 promoter
SV40 ori
NeoR/KanR
HSV TK poly(A) signal
ori
1000
2000
3000
4000
5000
6000

F

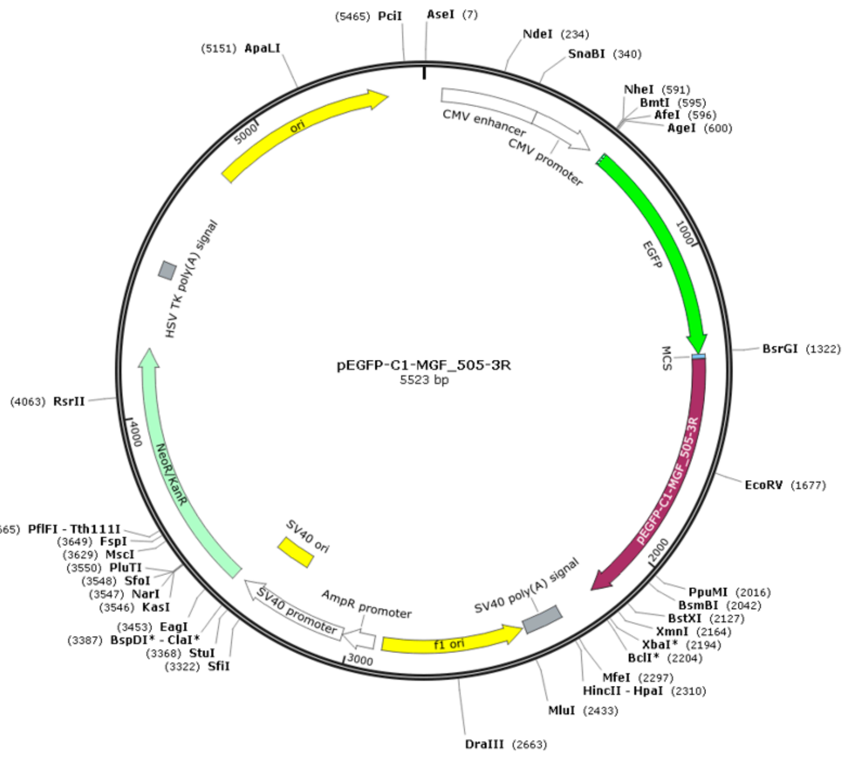

G

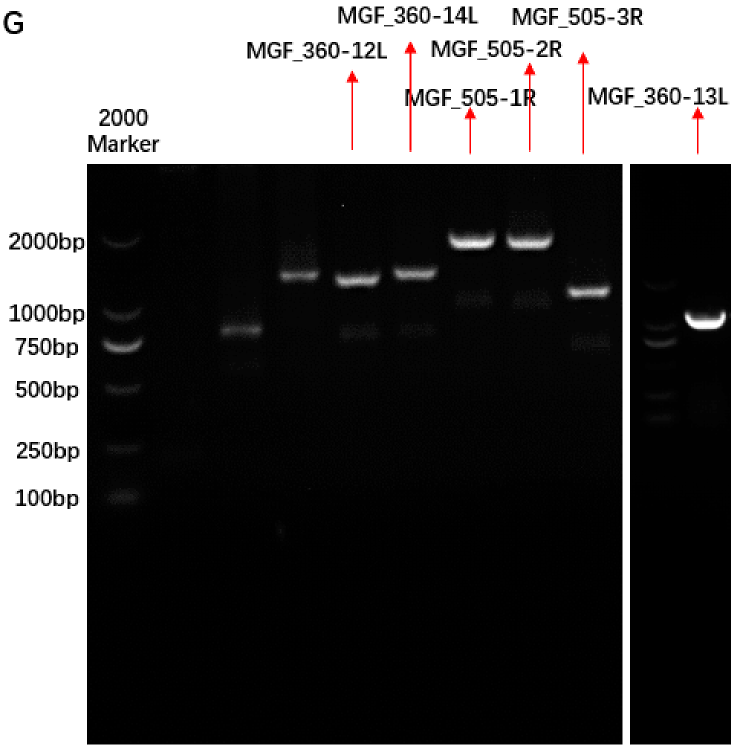

F i g u r e S2. Construct MGF360-505R plasmids. (**A–F**) Plasmids pCAGGs-MGF360-12L, pCAGGs-MGF360-13L, pCAGGs-MGF360-14L, pCAGGs-MGF505-1R, pCAGGs-MGF505-2R, pCAGGs-MGF505-3R were constructed using PCR and homologous recombination through target fragment and empty vector. (**G**) The constructed plasmid was verified by PCR and Sanger sequencing.

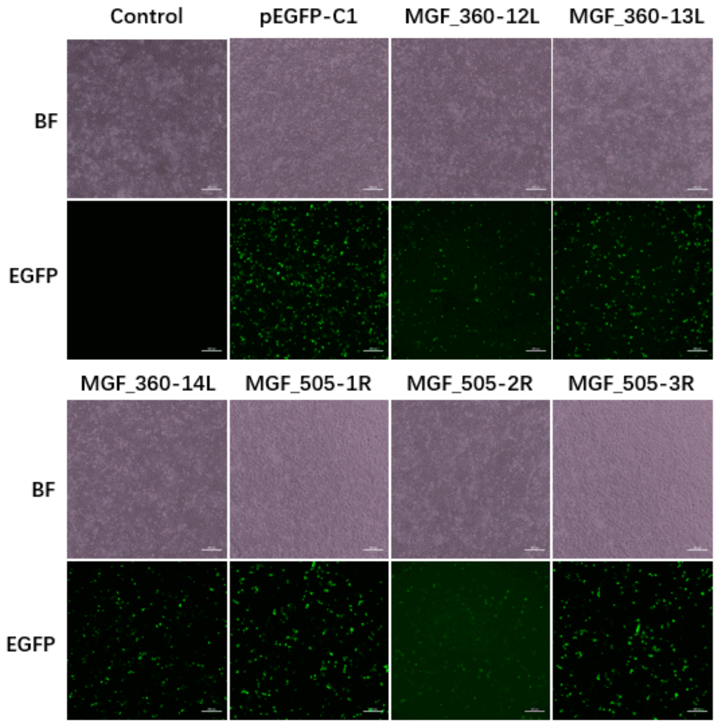

Figure S3. All of these plasmids, pCAGGs-MGF360-12L, pCAGGs-MGF360-13L, pCAGGs-MGF360-14L, pCAGGs-MGF505-1R, pCAGGs-MGF505-2R, pCAGGs-MGF505-3R, were successfully expressed in PK-15 cells. The green fluorescence observed under an inverted fluorescence microscope is the expression of MGF protein.

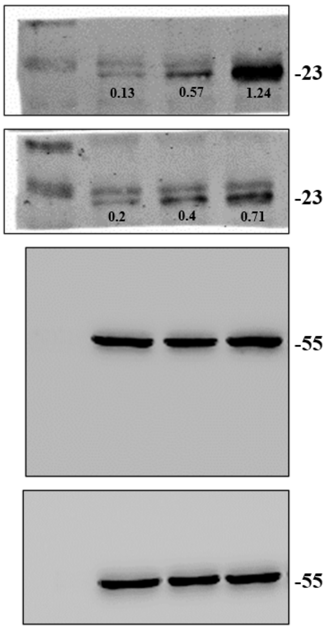

Figure S4. Detection of GZ201801-ASFV, ΔMGF360-505R-ASFV, ΔCD2v-ASFV, and ΔCD2v/ΔMGF360-505R-ASFV in primary PAMs. Western blotting was used to detect the expression of p30 protein. PAMs were either infected with GZ201801-ASFV , ΔMGF360-505R-ASFV, ΔCD2v-ASFV, and ΔCD2v/ΔMGF360-505R-ASFV at a MOI of 0.1.

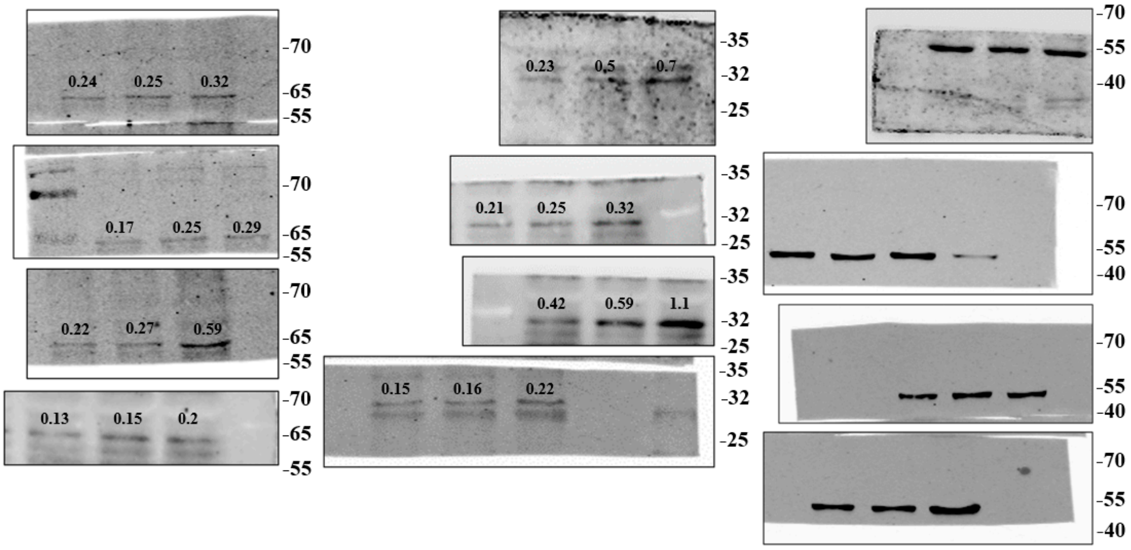

Figure S5. NF-κB signaling pathway analysis after ASFV infection. Western blotting was used to measure the expression of PP65 and PIκB protein at 3, 6, and 12 h in each group of GZ201801-ASFV-, ΔMGF360-505R-ASFV-, ΔCD2v-ASFV-, and ΔCD2v/ΔMGF360-505R-ASFV-infected PAMs. Expression of tubulin was used as a positive control. PAMs were either infected with GZ201801-ASFV or ΔCD2v/ΔMGF360-505R-ASFV at a MOI of 0.1.
